# Complexity of understanding the role of dietary and erythrocyte docosahexaenoic acid (DHA) on the cognitive performance of school-age children

**DOI:** 10.1093/cdn/nzac099

**Published:** 2022-06-16

**Authors:** Kelly A Mulder, Roger A Dyer, Rajavel Elango, Sheila M Innis

**Affiliations:** Department of Pediatrics, Faculty of Medicine, University of British Columbia, Vancouver, BC, Canada; Department of Pediatrics, Faculty of Medicine, University of British Columbia, Vancouver, BC, Canada; Analytical Core for Metabolics and Nutrition (ACMaN), BC Children's Hospital Research Institute, Vancouver, BC, Canada; Department of Pediatrics, Faculty of Medicine, University of British Columbia, Vancouver, BC, Canada; School of Population and Public Health, University of British Columbia, Vancouver, BC, Canada; Department of Pediatrics, Faculty of Medicine, University of British Columbia, Vancouver, BC, Canada

**Keywords:** DHA intake, DHA status, dietary assessment, cognitive performance, brain development, children

## Abstract

**Background:**

Early childhood is a period of rapid brain development, with increases in synapses rich in the omega-3 (ω-3) fatty acid, DHA (22:6ω-3) continuing well beyond infancy. Despite the importance of DHA to neural phospholipids, the requirement of dietary DHA for neurodevelopment remains unclear.

**Objectives:**

The aim was to assess the dietary DHA and DHA status of young children, and determine the association with cognitive performance.

**Methods:**

This was a cross-sectional study of healthy children (5–6 y), some of whom were enrolled in a follow-up of a clinical trial (NCT00620672). Dietary intake data (*n *= 285) were assessed with a food-frequency questionnaire (FFQ) and three 24-h recalls. Family characteristics were collected by questionnaire, and anthropometric data measured. Venous blood was collected, cognitive performance assessed using several age-appropriate tools including the Kaufman Assessment Battery for Children. The relation between dietary DHA, RBC DHA, and child neurodevelopment test scores was determined using Pearson's correlation or Spearman's rho, and quintiles of test scores compared by Mann–Whitney *U* test.

**Results:**

Child DHA intakes were highly variable, with a stronger association between RBC DHA and DHA intake assessed by FFQ (rho = 0.383, *P *< 0.001) compared with one or three 24-h recalls. Observed ethnic differences in DHA intake status as well as neurodevelopmental test scores led to analysis of the association between DHA intake and status with neurodevelopment test scores for White children only (*n* = 190). Child RBC DHA status was associated with neurodevelopment test scores, including language (rho = 0.211, *P *= 0.009) and short-term memory (rho = 0.187, *P *= 0.019), but only short-term memory was associated with dietary DHA (rho = 0.221, *P *= 0.003).

**Conclusions:**

Child RBC DHA but not dietary DHA was associated with multiple tests of cognitive performance. In addition, DHA intake was only moderately associated with RBC DHA, raising complex questions on the relation between diet, DHA transfer to membrane lipids, and neural function.

## Teaser

In children aged 5–6 y red blood cell (RBC) docosahexanoic acid (DHA), but not dietary intake of DHA, is associated with multiple tests of cognitive performance, emphasizing the complexity of DHA metabolism.

## Introduction

The ω-3 fatty acid DHA (22:6ω-3) is an essential component of neural phospholipids, accumulating in neural tissue during development. Rapid DHA accretion in the brain begins in utero and continues until at least 2y (1), suggesting that a postnatal source of ω-3 fatty acids to supply the developing brain with DHA is important. DHA can be synthesized endogenously from α-linolenic acid (ALA; 18:3ω-3) via fatty acid desaturase (FADS) and elongase (ELOVL) enzymes (2). However, a high dietary intake of linoleic acid (LA; 18:2ω-6) may antagonize the metabolism of ALA to DHA, through competition of the FADS and ELOVL enzymes (3–6). DHA can also be consumed in the diet in the form of animal tissue lipids, particularly aquatic organisms, with smaller amounts in eggs and poultry, and very small amounts in ruminants, depending on the tissue (7). Decreased brain DHA may therefore result from inadequate ω-3 and/or excessive ω-6 fatty acid intakes but is likely to occur only when the dietary DHA is very limited. Thus, dietary DHA should support brain DHA needs, regardless of dietary ω-6 fatty acid or ALA intakes.

Studies in animals and in human infants have shown that lower brain DHA is accompanied by an increase in brain 22:4ω-6 and 22:5ω-6 (8–11), which, in animals, is associated with deficits in neural function (8, 11–15). In humans, several observational and intervention studies of DHA in maternal gestation alone or including gestation and the first few weeks after birth have reported no effect (16–18) or positive effects on neural and visual function of infants and children (19–29). However, the DHA intake or blood lipid status of children has also been shown to be associated with central nervous system (CNS) function, including learning ability, language, and nonverbal intelligence, with some studies showing a benefit of DHA supplementation (30–38). This raises the possibility that the pre- and/or postnatal DHA supply has the potential to impact CNS development when assessed in childhood.

Although brain growth is rapid, reaching approximately 70% of adult weight by 1 y of age (39), early childhood is an important period of brain development, with a substantial increase in synapses rich in DHA continuing until 6–7 y of age (40). This suggests that brain DHA availability, and hence diet, may be important for the morphological and organizational changes in brain development continuing in early childhood. However, the dietary, genetic, and other variables that impact DHA transfer to the brain and the effect on neurodevelopment remain unclear.

Associations between maternal and child dietary intakes complicate interpretation of epidemiologic studies on DHA intake or DHA in blood lipids during pregnancy or childhood on CNS-related outcomes in children (41–44). For example, it has been reported that the fish intake of mothers and children 8–11 y of age was not different, and that the mothers’ prenatal fish intake was lower than their postnatal intake (43). More recently, we demonstrated that maternal RBC and dietary DHA during early and late gestation was associated with both the RBC and dietary DHA of the child at 5–6 y of age (45). Adding complexity are studies showing that the DHA supply in the first year of life also has a beneficial impact on neurodevelopment measured in infancy (46, 47) and lasting at least into childhood (48, 49). Taken together with the data that increased DHA intake in childhood can improve neural function in some children (33, 34, 36, 49, 50), it may be possible to overcome or correct effects of inadequate DHA in early development. However, further work is required to identify the timing of critical periods of development requiring an adequate DHA supply.

As previously noted, research on the needs of children for ω-6 and ω-3 fatty acids, specifically DHA for CNS development, knowledge of their dietary intakes, and implications for ω-3 fatty acid inadequacy is limited. Therefore, the objective of this study was to assess the DHA intake and status (RBC DHA), and determine the association with neurodevelopment test scores in a group of young children (5–6 y) in Vancouver, BC, Canada.

## Methods

### Study design

This study enrolled children aged 5 y and 9 mo ± 4 wk (5.75 y) with a parent from the community in Vancouver, BC: these participants had no previous involvement in our studies. A group of 98 children with a parent who had participated in the study of DHA during gestation (23) (ClinicalTrial.gov: NCT00620672) was also studied at 5.75 y (follow-up group) (45). In our previous study, pregnant women were randomly assigned to receive 400 mg DHA/d or a placebo from 16 wk of gestation until infant delivery. The infants were subsequently assessed at regular intervals up to 18 mo with a long-term follow up at 5.75 y (23, 45). For the present study, inclusion criteria in both groups included term gestation; singleton birth; no known conditions that may interfere with dietary intake, metabolism, growth, or development; and the parental ability to communicate in English. All participants were assigned a unique, computer-generated code upon enrollment, which was used for all study documents and blood samples. The primary investigator and the study assistant were responsible for the security of identifiable data.

The present study was conducted according to guidelines laid down in the Declaration of Helsinki. All procedures and forms involving human subjects in this study were approved by the Committee for Ethical Review of Research Involving Human Subjects at the University of British Columbia and the British Columbia Children's and Women's Hospital (H09-02921 and H09-01633). Written informed consent was obtained from a parent for each child before enrollment. The consent form stated that “Participants may not directly benefit from involvement in the study.” Both the cross-sectional and follow-up studies involved only a single visit to the research center, and all travel expenses (e.g., parking, bus fare) were reimbursed. Parents/caregivers were allowed to choose to receive up-to-date nutrition education materials and a small age-appropriate gift for their child. All participants received a study conclusion letter, and during study recruitment, a newsletter was shared with those participants who requested it, which shared updates, progress, and initial results.

### Participant characteristics

Sociodemographic and family information, including parental age, ethnicity, income, and the number of adults and children in the home, was collected by questionnaire. We assessed parental IQ with the Test of Non-Verbal Intelligence (TONI)-III, which assesses aptitude, abstract reasoning, and problem solving, and does not depend on formal education achieved (51). Child weight and height were measured in light clothing; and weight-for-age, height-for-age, and BMI-for-age *z* scores were calculated using the WHO Anthroplus anthropometric calculator (version 1.0.4).

### Dietary assessment

The children's dietary intake over the previous 4 wk was estimated by interview with a parent using a quantitative food-frequency questionnaire (FFQ). The FFQ included all foods and beverages consumed, with questions specific to fish type, cooking methods, and the use of fortified foods and supplements. Food models and measuring utensils were used to assist with estimation of actual portion sizes consumed by the children. Three 24-h recalls were also administered, using the 5-pass review technique (52). The first 24-h recall was given in person, and two 24-h recalls were conducted by telephone on random days over the following 2 wk. The children's dietary intake of all nutrients relevant to this research was quantified using nutrient analysis software (ESHA Food Processor SQL, ESHA Research, version 10.10.0.0), using Canadian foods, and checking the fatty acid composition of all foods and beverages for accuracy, modifying, if necessary, based on labels or laboratory analysis of the product.

### Child neurodevelopment

Child neurodevelopment was assessed using several age-appropriate and standardized tests. Assessments were conducted in a clinical testing room at the BC Children's Hospital Research Institute, which contains a 1-way mirror to enable the parent(s) to view the child from an adjacent room. All tests were administered on the same day by 1 of 2 individuals trained in child psychometric testing, with assessment and training led by a trained research assistant with a Master of Arts (MA) in child development. The tests used were the Kaufman Assessment Battery for Children (KABC), 2nd edition (KABC-II), which assesses the IQ of children aged 2–12 y and provides a scaled score for multiple domains of cognitive ability, including sequential (short-term memory) and simultaneous (visual processing) processing, and learning ability (long-term storage and retrieval) (53). The sum of these 3 domains provides a composite Mental Performance Index (MPI) score (general mental processing ability) (53). Long-term memory was assessed with the KABC Delayed Recall scale. Language development was assessed using the Peabody Picture Vocabulary Test (PPVT) (54), visual-motor integration was assessed with the Beery–Buktenica Developmental Test of Visual Motor Integration (Beery) (55), and attention and impulsivity were assessed using the Test of Variables of Attention (TOVA) (56).

### Biochemical analysis

Venous blood (6 mL) was collected from the children by a certified phlebotomist, with EDTA as the anticoagulant. The plasma and buffy coat were separated by centrifugation (2500 × *g*,  10 min,  4°C), the RBC washed with saline and EDTA and centrifuged twice, and the RBC pellet was stored at –80°C until analysis. RBC fatty acids were extracted and quantified by routine gas-liquid chromatography (57).

### Data analysis

All data were checked for normality using a 1-sample Kolmogorov-Smirnov test. Child and family characteristics were summarized for each group of children using descriptive statistics. Child and family characteristics, dietary and RBC fatty acids, and child neurodevelopment test scores were compared between the follow-up and cross-sectional children using chi-square, Student's *t* test, or Mann–Whitney *U* test, as appropriate.

Dietary DHA intakes estimated by FFQ, single 24-h recall, and the average of three 24-h recalls were compared using ANOVA, with Bonferroni correction for multiple comparisons. The relation between dietary DHA for intakes calculated using the 3 approaches and RBC DHA was determined using Pearson's correlation or Spearman's rho. The data on dietary supplements were not included in the analysis, as many parents were not able to recall the brand, source, and/or amount of ω-3 fatty acids contained in the supplement. However, only approximately 20% of the children were reported to have taken ω-3 supplements, and of these with complete data, the majority of children took supplements containing a small amount of DHA (∼25 mg).

The potential association of dietary and RBC fatty acids with child neurodevelopment test scores was determined using partial Pearson's correlation coefficients, adjusting for child or family characteristics found to be associated with child test scores. The relation between an essential nutrient and neurological function is curvilinear; neither the minimum nor optimum DHA intake or biomarker equivalent of adequate DHA for the brain has been established. Therefore, we sought to determine if dietary and RBC DHA and related ω-6 fatty acids differed between children with the highest and lowest scores for each neurodevelopment test. To achieve this, the mean or median dietary and RBC fatty acids of interest were compared for children in the highest and lowest quintiles for each test using Student's *t* test or Mann–Whitney *U* test, as appropriate. Data analysis was conducted using IBM SPSS Statistics (version 20.0.0, 2011; IBM Corporation), with significance set at *P* < 0.05.

## Results

A total of 98 and 187 children, all aged 5.75 y, were enrolled in the follow-up and cross-sectional studies, respectively (total *n* = 285). A single 24-h recall was analyzed for 272 (95%) children, three 24-h recalls were analyzed for 259 (91%) children, and the FFQ was analyzed for 280 (98%) children. Blood samples were obtained from 245 (86%) children. Cognitive tests were completed for >90% of the children, with incomplete tests for PPVT (*n* = 14); KABC Sequential (*n* = 10), Learning (*n* = 15), Simultaneous (*n* = 5), MPI (*n* = 24), Delayed Recall (*n* = 21); and Beery (*n* = 1). One exception was the TOVA, which was incomplete for approximately 20% of children (*n* = 56). The high number of incomplete assessments may have affected the validity of these results in our study and we therefore chose not to include the TOVA scores.

Comparison of characteristics between the follow-up and cross-sectional groups showed that parents of children in the follow-up group had slightly higher TONI scores (36.1 ± 6.86 and 34.2 ± 6.84, respectively; *P *= 0.014), and a greater percentage had 3 or more rather than 1 or 2 children (23.7% and 15.1%, respectively; *P *= 0.021) (**Table 1**). There were no significant differences between the follow-up and cross-sectional groups for ethnicity, number of adults in the home, child sex, exclusive human-milk feeding for >3 mo, or children's BMI-for-age (*P *> 0.05; Table 1). For the entire group of children, 50.9% were boys, 81.6% of the children lived with 2 adults, 85.6% had been human-milk fed at least 3 mo, and the mean (SD) BMI *z* score at 5.75 y was 0.15 (0.96). In addition, 66.7% of the children were White, 15.8% were Chinese, and 17.5% were of another ethnicity, including East Indian/South Asian (7.0%), other Asian (4.2%), Hispanic (2.5%), African (1.1%), Persian (1.1%), Jewish (0.7%), First Nations (0.4%), Egyptian (0.4%), and Mediterranean (0.4%) (ethnicity was self-reported). Thus, due to the small number of children of other ethnicities (*n* = 50), subsequent analyses addressing a need for adjustment by ethnicity used only data for children of White (*n* = 190) and Chinese (*n* = 45) ethnicity.

**TABLE 1:** Family and child characteristics for all children and for the follow-up and cross-sectional groups^1^

	All (*n* = 285)	Follow-up (*n* = 98)	Cross-sectional (*n* = 187)	*P*
White/Chinese/other,^2^ %	66.7/15.8/17.5	74.5/11.2/14.3	62.6/18.2/19.2	0.131
Parental TONI IQ, mean ± SD	34.8 ± 6.90	36.1 ± 6.86	34.2 ± 6.84	0.014
Adults in home (2/1/>2), %	81.6/8.83/9.54	83.5/5.15/11.3	80.6/10.8/8.60	0.350
Children in home (2/1/>2), %	62.5/19.4/18.0	64.9/11.3/23.7	61.3/23.6/15.1	0.021
Child sex (boys/girls), %	50.9/49.1	45.9/54.1	53.5/46.5	0.262
Child human milk-fed >3 mo, %	85.6	89.8	83.4	0.159
Child BMI-for-age, *z* score, mean ± SD	0.15 ± 0.96	0.08 ± 0.99	0.18 ± 0.94	0.427

1 TONI, Test of Non-Verbal Intelligence.

2 Ethnicity was by self-report and for “other” is: East Indian/South Asian (n = 20), Other Asian (n = 12), Hispanic (n = 7), African (n = 3), Persian (n = 3), Jewish (n = 2), and *n* = 1 for each of First Nations, Egyptian, and Mediterranean,

There were no differences between the follow-up and cross-sectional groups’ dietary intake assessed using the FFQ, 24-h recall prior to blood collection, or the average of three 24-h recalls for total energy, protein, carbohydrate, and total fat. In addition, there were no differences in LA, ALA, EPA (20:5ω-3), or DHA intakes (*P *> 0.05), and only a small statistical difference in arachidonic acid (ARA; 20:4ω-6) intake assessed by FFQ (**Supplemental Table 1**). Significant differences were found for some RBC fatty acids between the follow-up and cross-sectional groups of children, but not the RBC DHA (**Supplemental Table 2**). We also observed some minor differences in neurodevelopmental test scores, with higher scores in the follow-up cohort on the KABC scales of Learning and MPI compared with the cross-sectional cohort (**Supplemental Table 3**). As the differences between the groups were small, the 2 cohorts of children were combined for the remaining analyses.

The dietary intake of energy, macronutrients, and ω-3 and ω-6 fatty acids for all children was higher when estimated by FFQ than with one or three 24-h recalls. For total energy, the median (2.5–97.5 percentile) intake assessed by FFQ was 1785 (1035–3033) kcal/d, but 1495 (848–2563) and 1489 (926–2289) kcal/d when using one and three 24-h recalls, respectively (*P *< 0.001) (**Supplemental Table 4**). Similarly, total fat, carbohydrate, and protein intakes were also higher when assessed by FFQ than one or three-day 24-h recall (*P* < 0.05), with no difference in macronutrient intake when intake was calculated per kilocalorie (Supplemental Table 4). Thus, the mean ranges as a percentage of total energy were 32.7–33.3% total fat, 53.1–53.9% carbohydrate, and 15.8–16.1% protein for all 3 dietary intake approaches. The intakes of LA, ARA, and ALA were higher when assessed by FFQ, again with no difference in intake among the methods when expressed as g/1000 kcal (**Table 2**). However, EPA and DHA intakes were higher when assessed by FFQ than the one and three 24-h recalls, expressed as either mg/d or mg/1000 kcal (Table 2).

**TABLE 2:** Children's ω-3 and ω-6 fatty acid intake assessed using FFQ and one or three 24-h recalls^1^

	FFQ (*n* = 280)		1 × 24-h Recall (*n* = 272)		3 × 24-h Recall (*n* = 259)		
	Mean ± SD	Median (2.5–97.5th percentile)	Mean ± SD	Median (2.5–97.5th percentile)	Mean ± SD	Median (2.5–97.5th percentile)	*P* ^2^
ω-3 Fatty acids							
DHA, mg/d	77.7 ± 80.6	52.9 (4.61–327)	48.2 ± 112	12.2 (0.00–367)	57.7 ± 108	19.5 (1.20–328)	<0.001
DHA, mg/1000 kcal	41.3 ± 39.4	30.9 (2.53–156)	32.9 ± 76.0	8.23 (0.00–292)	37.1 ± 62.1	13.0 (0.63–206)	<0.001
EPA, mg/d	45.5 ± 56.9	26.5 (0.90–248)	25.4 ± 79.0	2.70 (0.00–274)	31.4 ± 74.9	4.20 (0.05–216)	<0.001
EPA, mg/1000 kcal	24.1 ± 27.8	16.4 (0.43–108)	17.6 ± 54.3	1.90 (0.00–219)	20.3 ± 44.4	2.57 (0.00–146)	<0.001
ALA, g/d	1.27 ± 0.61	1.15 (0.51–2.85)	1.14 ± 0.90	0.87 (0.25–3.61)	1.04 ± 0.56	0.92 (0.39–3.12)	<0.001
ALA, g/1000 kcal	0.68 ± 0.26	0.62 (0.37–1.25)	0.73 ± 0.54	0.58 (0.22–2.54)	0.69 ± 0.36	0.61 (0.32–1.78)	0.292
ω-6 Fatty acids							
LA, g/d	9.70 ± 4.25	8.79 (3.64–20.6)	8.64 ± 5.84	7.25 (2.04–24.0)	8.00 ± 3.49	7.25 (3.07–17.7)	<0.001
LA, g/1000 kcal	5.14 ± 1.43	4.88 (2.91–8.72)	5.42 ± 2.66	4.91 (1.88–11.6)	3.29 ± 1.35	3.03 (1.47–7.09)	0.888
ARA, mg/d	83.4 ± 54.5	68.6 (12.6–210)	77.3 ± 71.6	51.2 (0.85–270)	76.5 ± 54.9	63.3 (7.05–220)	0.002
ARA, mg/1000 kcal	44.9 ± 25.5	39.3 (6.96–109)	50.3 ± 45.6	34.2 (0.64–159)	49.4 ± 33.2	42.0 (4.27–133)	0.142

1 ALA, ɑ-linolenic acid; ARA, arachidonic acid; FFQ, food-frequency questionnaire; LA, linoleic acid.

2 Data by the 3 dietary approaches analyzed by Kruskal-Wallis test for non-normal distributions.

**FIGURE 1:**
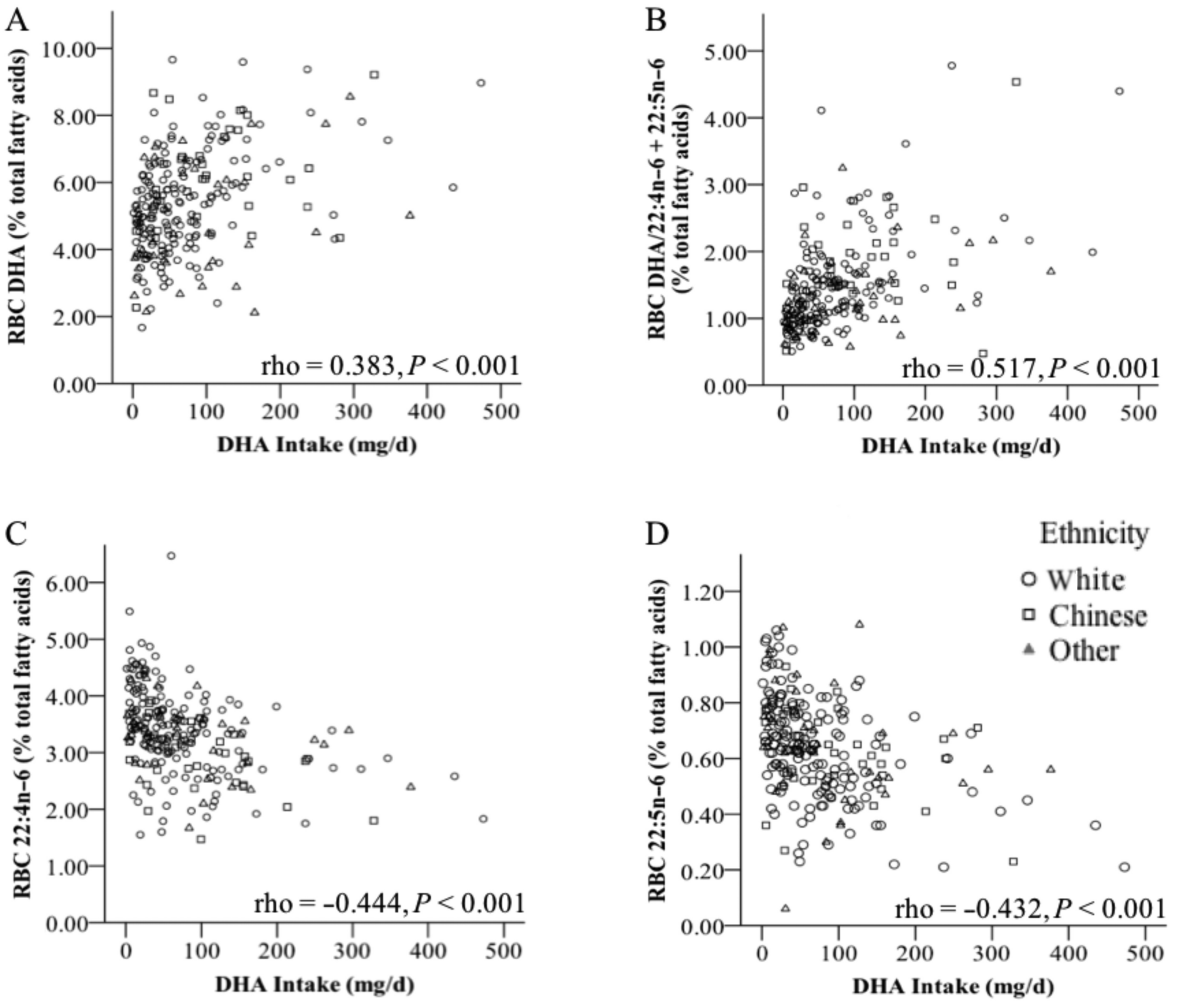
Scatterplots of the association between DHA intake and RBC fatty acids. The data were analyzed using Spearman's rank correlation coefficient between DHA intake and RBC DHA (A), DHA/22:4ω-6 + 22:5ω-6 ratio (B), 22:4ω-6 (C), and 22:5ω-6 (D) for children of White (O, *n* = 160), Chinese (☐, *n* = 37), and other ethnicity (Δ, *n* = 40).

The association between DHA intake and relevant RBC fatty acids was stronger using dietary intake data collected using the FFQ rather than one or three 24-h recalls (**Supplemental Table 5**). DHA intake assessed by FFQ was positively related to the RBC DHA (rho = 0.383, *P *< 0.001) and EPA (rho = 0.457, *P *< 0.001), with an inverse association to RBC 20:4ω-6 (rho = –0.244, *P *< 0.001), 22:4ω-6 (rho = –0.444, *P *< 0.001), and 22:5ω-6 (rho = –0.432, *P *< 0.001). Notably, DHA intake showed the greatest association with RBC DHA/22:4ω-6 + 22:5ω-6 (rho = 0.517, *P *< 0.001). However, scatterplots of DHA intake and RBC ω-3 and ω-6 fatty acids show high individual variability, particularly at DHA intakes <175 mg/d (**Figure 1**). The group numbers appear to fit a predicted pattern of higher DHA status with higher DHA intake, although individual children's status in relation to diet is varied.

Children of Chinese ethnicity had significantly higher intakes of DHA (*P *= 0.005), EPA (*P *= 0.013), and ARA (*P *< 0.001) compared with White children (**Supplemental Table 6**). There were no differences between the Chinese and White children for dietary intakes of LA (*P *= 0.105) or ALA (*P *= 0.091). We found no other child or family characteristic collected in our study associated with ω-3 and ω-6 fatty acid intake (*P *> 0.05). Consistent with the dietary DHA intake, the RBC DHA was also highest in children of Chinese descent compared with White children, with a mean (SD) of 6.06% (1.42%) and 5.38% (1.52%) Total Fatty Acids, respectively, (*P *= 0.013) (**Supplemental Table 7**). RBC LA was also higher, but 22:4ω-6 was lower in Chinese children compared with children of a Caucasian background. The association between DHA intake and RBC fatty acids also suggests possible differences by ethnicity, with stronger associations observed when ethnicity is included as a covariate in the adjusted model (**Table 3**). A significant positive association was observed between DHA intake and RBC DHA (rho = 0.394, *P* < 0.001), and more notably the association between DHA intake and RBC 20:5ω-3 (rho = 0.485, *P* < 0.001), both adjusted for ethnicity. Notably, DHA was negatively associated with RBC 22:4ω-6 (rho = –0.444, *P* < 0.001, unadjusted) and the association weakened when ethnicity was included (rho = –0.276, *P* < 0.001, adjusted). Finally, the strongest association was observed between DHA intake and RBC DHA/22:4ω-6 + 22:5ω-6 (rho = 0.517, *P* < 0.001, unadjusted; rho = 0.510, *P* < 0.001, adjusted).

**TABLE 3:** Association between DHA intake and RBC fatty acids^1^

		DHA intake (FFQ)	
		All	
RBC	rho or *P*	Unadjusted^2^ (*n* = 239)	Adjusted^3^ (df = 196)
ω-3 Fatty acids			
DHA	rho	0.383	0.394
	*P*	<0.001	<0.001
22:5ω-3	rho	–0.190	–0.073
	*P*	0.003	0.308
20:5ω-3	rho	0.457	0.485
	*P*	<0.001	<0.001
18:3ω-3	rho	0.016	0.065
	*P*	0.811	0.361
ω-6 Fatty acids			
22:5ω-6	rho	–0.432	–0.423
	*P*	<0.001	<0.001
22:4ω-6	rho	–0.444	–0.276
	*P*	<0.001	<0.001
20:4ω-6	rho	–0.244	–0.261
	*P*	<0.001	0.001
18:2ω-6	rho	–0.021	–0.095
	*P*	0.747	0.183
DHA/22:4ω-6 + 22:5ω-6	rho	0.517	0.510
	*P*	<0.001	<0.001

1 FFQ, food-frequency questionnaire.

2 Values are Spearman's correlation coefficients for the association between DHA intake and RBC fatty acids. ^3^Values are partial correlations adjusted for child ethnicity.

**FIGURE 2:**
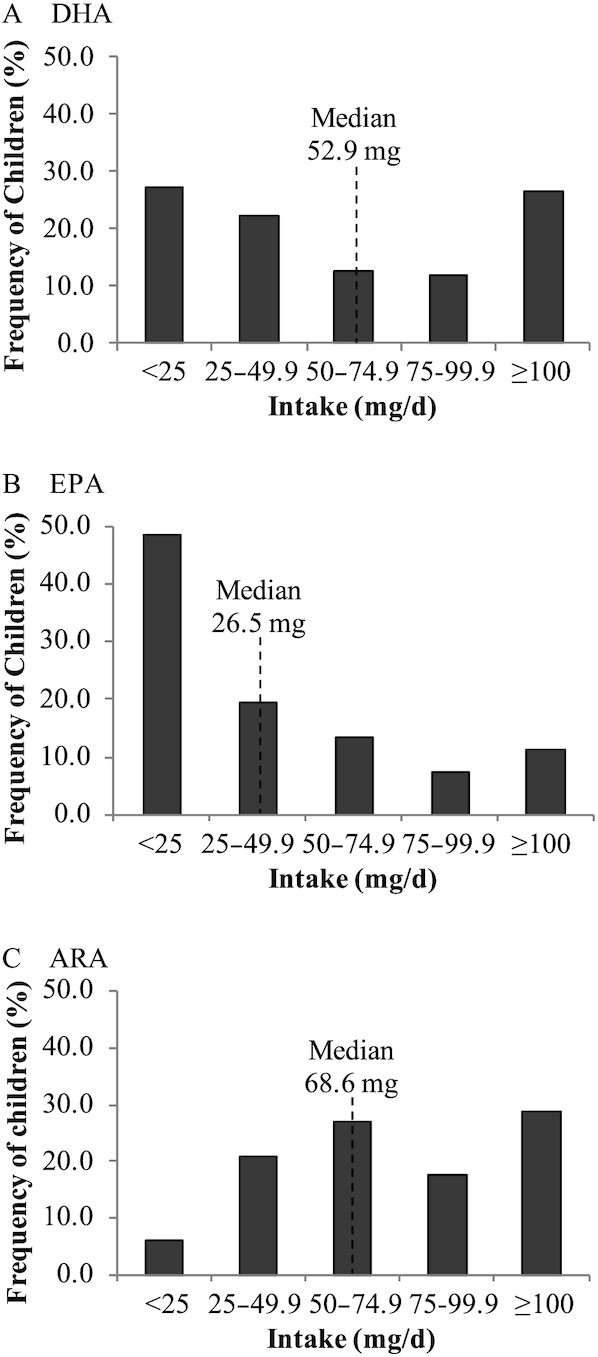
Frequency distributions of DHA, EPA, and ARA intake for all children. Bars indicate the percentage of children with intakes in increments of 25 mg/d for DHA (A), EPA (B), and ARA (C) for all children (*n*  = 280).

**FIGURE 3:**
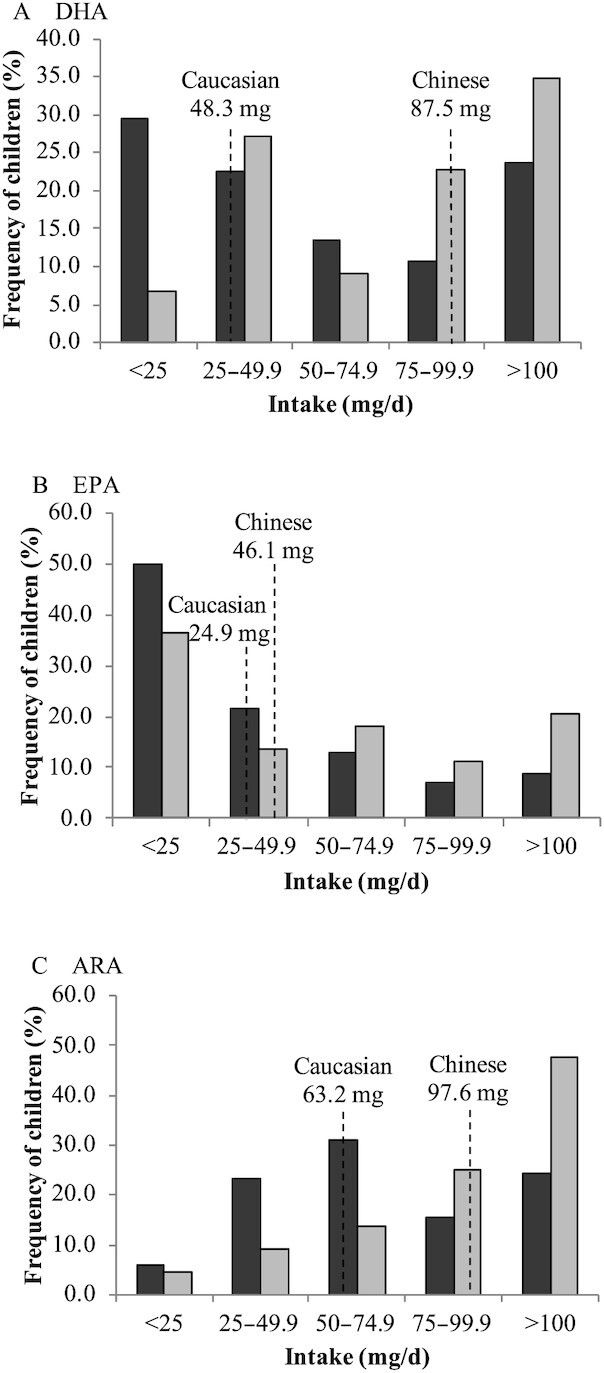
Frequency distributions of DHA, EPA, and ARA intake for White and Chinese children. Bars indicate the percentage of children with intakes in increments of 25 mg/d for DHA (A), EPA (B), and ARA (C) for White (dark-gray, *n* = 162) and Chinese (light-gray, *n*  = 37) children. The dashed line indicates the median intake of the respective fatty acid.

The intakes of DHA, EPA, and ARA for all children were highly skewed, with a median (2.5–97.5 percentile) of 52.9 (4.61–327), 26.5 (0.90–248), and 68.6 (12.6–210) mg/d assessed by FFQ. **Figure 2** shows the frequency of intakes of DHA, EPA, and ARA in increments of 25 mg/d. The intake of DHA appears bimodal, with more individuals at the tails of the distribution than clustered around the median. For example, 53.5% of the children had DHA intakes at the tails of the distribution, ≤25 and ≥100 mg/d, while 46.4% had intakes clustered within 25 mg above and below the median-containing interval. In contrast, for EPA and ARA intakes, 81.1% and 65% of the children had intakes clustered within 25 mg above and below the median-containing interval. **Figure 3** shows the frequency of DHA, EPA, and ARA intakes for children of White and Chinese backgrounds separately. Figure 3 highlights that the shape of the intake distributions differs between White and Chinese children, with the differences most pronounced for DHA intakes, with more White than Chinese children with DHA intakes ≤25 mg/d.

Differences in developmental test scores were also present among children of White and Chinese ethnicity (**Supplemental Table 8**). Children of Caucasian descent had higher [median (IQR)] scores on the PPVT [121 (112–132)] compared with children of Chinese descent [108 (90.5–121)] (*P *< 0.001). In contrast, children of White background had lower scores on the Beery (*P *= 0.004) and KABC Learning (*P *= 0.022), Simultaneous (*P *= 0.009), and MPI (*P *= 0.050) scales compared with children of Chinese ethnicity. No significant differences among ethnicities were found for scores on the KABC Sequential and Delayed Recall scales (*P *> 0.05).

Due to the relatively smaller sample size of Chinese and other ethnicities and the potential interaction with diet, RBC fatty acids, and test scores, we assessed the relation between the dietary and RBC relevant fatty acids and test scores for White children only (*n* = 190). For White children, only the KABC Sequential scale was associated with dietary fatty acid intake, with a positive association with DHA (rho = 0.227, *P *= 0.002) and EPA (rho = 0.258, *P *= 0.001) (**Table 4**). Similarly, there were no differences in dietary DHA, EPA, or ARA between children in the highest and lowest quintiles for the PPVT, Beery, KABC Learning, Simultaneous, MPI, and Delayed Recall scales (data not shown). DHA intake was related to the KABC Sequential scale, with a higher [median (IQR)] intake among children in the highest quintile [69.9 (13.1–48.9) mg/d] compared with the lowest quintile of scores [24.1 (13.1–48.9) mg/d] (*P *< 0.001) (**Supplemental Table 9**). EPA intake was also higher in the highest quintile of KABC Sequential scale scores (*P *< 0.001). Notably, dietary EPA and DHA are tightly correlated in our group (rho = 0.969, *P *< 0.001) because of fish intake, which is high in both EPA and DHA.

**TABLE 4:** Associations between dietary DHA, EPA, and ARA and neurodevelopment test scores for White children^1^

		DHA, mg/d		EPA, mg/d		ARA, mg/d	
	*n*	rho^2^	*P*	rho^2^	*P*	rho^2^	*P*
PPVT	174	0.137	0.072	0.128	0.093	–0.013	0.861
Beery	184	0.051	0.489	0.053	0.471	–0.043	0.558
KABC-II							
Sequential	176	0.221	0.003	0.252	0.001	0.048	0.528
Learning	173	0.029	0.702	0.016	0.839	0.045	0.556
Simultaneous	183	–0.013	0.861	–0.005	0.943	–0.050	0.502
MPI	169	0.044	0.566	0.061	0.430	–0.020	0.800
Delayed Recall	171	–0.027	0.723	–0.051	0.510	0.015	0.843

1 ARA, arachidonic acid; Beery, Beery–Buktenica Developmental Test of Visual-Motor Integration; KABC-II, Kaufman Assessment Battery for Children, 2nd edition; MPI, Mental Performance Index; PPVT, Peabody Picture Vocabulary Test.

2 Values are Spearman's correlation coefficients for the association between dietary fatty acids and neurodevelopmental test score for White children.

For White children, the RBC DHA was weakly, but positively, associated with scores on the PPVT (rho = 0.211, *P *= 0.008) and the KABC Sequential (rho = 0.182, *P *= 0.022) scale, with a nonsignificant trend for the MPI scale (rho = 0.149, *P = *0.067, *n* = 152) (**Table 5**). A significant positive association was found between RBC DHA/22:4ω-6 + 22:5ω-6 and scores on the PPVT (rho = 0.190, *P *= 0.018) and the KABC Sequential scale (rho = 0.231, *P *= 0.003). Next, we compared RBC fatty acids by quintile of test score for the White children (**Tables 6** and **7**). Children with test scores in the highest compared with the lowest quintile had RBCs with higher (mean ± SD %Total Fatty Acids) DHA for the KABC Sequential (4.95 ± 1.32 and 5.80 ± 1.51, *P *= 0.013) and MPI (5.07 ± 1.54 and 5.94 ± 1.50, *P *= 0.036) scales, with a close trend for the PPVT (5.26 ± 1.41 and 5.99 ± 1.41, *P *= 0.053). Similarly, the highest quintile compared with the lowest quintile had RBCs with higher [median, (IQR)] DHA/22:4ω-6 + 22:5ω-6 for the PPVT [1.58 (1.08–2.12) and 1.24 (0.96–1.48), *P *= 0.007] and KABC Sequential scale [1.46 (1.14–1.93) and 1.02 (0.83–1.35), *P *= 0.002], respectively. Lower RBC 22:5ω-6 was also found in the highest, compared with the lowest, quintile of children for the PPVT (0.70 ± 0.19 and 0.57 ± 0.17, *P *= 0.008) and KABC Sequential scale (0.72 ± 0.18 and 0.62 ± 0.20, *P *= 0.035) scores. There were no differences in RBC DHA and ω-6 fatty acids between children in the highest and lowest quintiles for the KABC Learning and Delayed Recall scales.

**TABLE 5:** Associations between RBC DHA, 22:5ω-6, and 22:4ω-6 and neurodevelopment test scores for White children^1^

		RBC fatty acids, % of total fatty acids							
		DHA		22:5ω-6		22:4ω-6		DHA/22:4ω-6 + 22:5ω-6	
	*n*	rho^2^	*P*	rho^2^	*P*	rho^2^	*P*	rho^2^	*P*
PPVT	153	0.211	0.009	–0.163	0.044	–0.081	0.321	0.190	0.018
Beery	164	0.102	0.192	–0.095	0.229	0.014	0.863	0.103	0.187
KABC-II									
Sequential	157	0.187	0.019	–0.163	0.042	–0.166	0.037	0.231	0.003
Learning	154	0.071	0.383	0.023	0.773	0.132	0.102	–0.042	0.603
Simultaneous	163	0.083	0.295	0.017	0.825	0.042	0.592	0.044	0.575
MPI	151	0.151	0.065	–0.022	0.790	0.020	0.804	0.093	0.253
Delayed Recall	150	–0.004	0.964	0.111	0.176	0.059	0.473	–0.049	0.552

1 Beery, Beery–Buktenica Developmental Test of Visual-Motor Integration; KABC-II, Kaufman Assessment Battery for Children, 2nd edition; MPI, Mental Performance Index; PPVT, Peabody Picture Vocabulary Test.

2 Values are Spearman's correlation coefficients for the association between RBC fatty acids and neurodevelopmental test score for White children.

**TABLE 6:** RBC fatty acids by quintiles of PPVT and Beery scores for White children^1^

	Quintiles of test scores^2^					
	1	2	3	4	5	*P* ^3^
PPVT, *n*	28	30	34	31	30	
DHA	5.26 ± 1.41	4.98 ± 1.52	5.30 ± 1.48	5.56 ± 1.66	5.99 ± 1.41	0.053
22:5ω-6	0.70 ± 0.19	0.64 ± 0.18	0.63 ± 0.17	0.66 ± 0.19	0.57 ± 0.17	0.008
22:4ω-6	3.44 ± 0.71	3.94 ± 0.86	3.49 ± 0.88	3.52 ± 0.77	3.20 ± 0.66	0.204
DHA/22:4ω-6 + 22:5ω-6^4^	1.38 ± 0.77	1.29 ± 0.64	1.39 ± 0.58	1.43 ± 0.74	1.69 ± 0.77	0.007
Beery, *n*	28	25	62	20	29	
DHA	5.24 ± 1.54	5.31 ± 1.58	5.28 ± 1.62	5.45 ± 1.22	5.77 ± 1.52	0.196
22:5ω-6	0.66 ± 0.16	0.70 ± 0.21	0.63 ± 0.17	0.64 ± 0.19	0.60 ± 0.17	0.152
22:4ω-6	3.37 ± 0.69	3.46 ± 0.91	3.44 ± 0.72	3.38 ± 0.67	3.48 ± 0.97	0.624
DHA/22:4ω-6 + 22:5ω-6^4^	1.41 ± 0.68	1.41 ± 0.74	1.38 ± 0.61	1.40 ± 0.40	1.60 ± 0.92	0.304

1 Beery, Beery–Buktenica Developmental Test of Visual-Motor Integration; PPVT, Peabody Picture Vocabulary Test.

2 Minimum–maximum of test scores in quintile 1–5 were as follows—for PPVT: 73–109, 111–115, 116–125, 126–233, 134–159; Beery: 11–14, 15, 16–17, 18, 19–23.

3 Quintiles 1 and 5 were compared by Student's *t* test unless otherwise indicated.

4 Quintiles 1 and 5 were compared by Mann–Whitney *U* test.

**TABLE 7:** RBC fatty acids by quintile of KABC-II scores for White children^1^

	Quintiles of test scores^2^					
KABC-II	1	2	3	4	5	*P* ^3^
Sequential, n	35	23	33	29	37	
DHA	4.93 ± 1.34	5.62 ± 1.18	5.49 ± 1.76	5.33 ± 1.72	5.80 ± 1.51	0.012
22:5ω-6	0.72 ± 0.18	0.64 ± 0.14	0.58 ± 0.15	0.63 ± 0.18	0.62 ± 0.20	0.029
22:4ω-6	3.66 ± 0.75	3.50 ± 0.63	3.23 ± 0.75	3.28 ± 0.78	3.33 ± 0.76	0.051
DHA/22:4ω-6 + 22:5ω-6^4^	1.24 ± 0.73	1.42 ± 0.48	1.54 ± 0.77	1.48 ± 0.76	1.56 ± 0.62	0.002
Learning, n	31	37	29	28	29	
DHA	5.09 ± 1.61	5.68 ± 1.71	5.37 ± 1.42	5.04 ± 1.49	5.71 ± 1.36	0.111
22:5ω-6	0.65 ± 0.18	0.62 ± 0.17	0.59 ± 0.20	0.64 ± 0.17	0.68 ± 0.18	0.585
22:4ω-6	3.33 ± 0.79	3.31 ± 0.69	3.23 ± 0.75	3.58 ± 0.77	3.66 ± 0.71	0.093
DHA/22:4ω-6 + 22:5ω-6^4^	1.37 ± 0.66	1.55 ± 0.76	1.56 ± 0.86	1.28 ± 0.55	1.39 ± 0.54	0.707
Simultaneous, n	34	24	35	32	38	
DHA	5.27 ± 1.32	5.26 ± 1.70	5.31 ± 1.95	5.39 ± 1.43	5.63 ± 1.30	0.248
22:5ω-6	0.63 ± 0.15	0.65 ± 0.17	0.63 ± 0.22	0.63 ± 0.20	0.66 ± 0.15	0.420
22:4ω-6	3.48 ± 0.72	3.28 ± 0.73	3.32 ± 0.82	3.36 ± 0.71	3.61 ± 0.87	0.492
DHA/22:4ω-6 + 22:5ω-6^4^	1.32 ± 0.40	1.50 ± 0.95	1.48 ± 0.82	1.46 ± 0.72	1.41 ± 0.55	0.710
MPI, n	28	28	42	26	27	
DHA	5.06 ± 1.56	5.52 ± 1.71	5.53 ± 1.55	4.94 ± 1.23	5.94 ± 1.50	0.037
22:5ω-6	0.65 ± 0.16	0.63 ± 0.17	0.61 ± 0.19	0.66 ± 0.18	0.65 ± 0.18	0.989
22:4ω-6	3.40 ± 0.72	3.37 ± 0.85	3.30 ± 0.72	3.46 ± 0.74	3.55 ± 0.75	0.450
DHA/22:4ω-6 + 22:5ω-6^4^	1.37 ± 0.79	1.49 ± 0.75	1.52 ± 0.75	1.30 ± 0.59	1.49 ± 0.54	0.147
Delayed Recall, n	24	26	38	33	29	
DHA	5.11 ± 1.36	5.49 ± 1.72	5.48 ± 1.78	5.58 ± 1.33	5.00 ± 1.40	0.765
22:5ω-6	0.65 ± 0.13	0.59 ± 0.20	0.62 ± 0.18	0.66 ± 0.18	0.69 ± 0.17	0.290
22:4ω-6	3.44 ± 0.71	3.33 ± 0.80	3.49 ± 0.93	3.44 ± 0.74	3.56 ± 0.58	0.502
DHA/22:4ω-6 + 22:5ω-6^4^	1.31 ± 0.51	1.56 ± 0.80	1.50 ± 0.94	1.44 ± 0.53	1.22 ± 0.46	0.500

1 KABC-II, Kaufman Assessment Battery for Children, 2nd edition; MPI, Mental Performance Index

2 Minimum–maximum of test scores in quintiles 1–5 were as follows—for KABC Sequential: 15–17, 18–19, 21–22, 23–24, 25–34; KABC Learning: 11–17, 18–20, 21–23, 24–26, 27–35; Simultaneous: 13–30, 31–32, 33–35, 36–38, 39–51; MPI: 39–68, 69–73, 74–82, 83–86, 87–113; Delayed Recall: 9–17, 18–19, 20–22, 23–25, 26–32.

3 Quintiles 1 and 5 were compared by Student's *t* test unless otherwise indicated.

4 Quintiles 1 and 5 were compared by Mann–Whitney *U* test.

## Discussion

In the present study, we addressed child DHA intake and RBC DHA, and their potential relation to neurodevelopment at age 5.75 y. The results suggest that child RBC DHA is associated with scores on some neurodevelopment tests assessed at 5.75 y, while dietary DHA was only associated with a test of short-term memory. Notably, variability in DHA intake and RBC DHA among the children was high, with a complex relation between DHA intake and RBC fatty acids. The association between DHA intake and RBC fatty acids was stronger with DHA intake assessed by FFQ compared with one or three 24-h recalls.

The DHA intakes of all children were skewed and bimodal, with a mean (median) intake of 77.7 (52.9) mg/d assessed by FFQ, with 48.2 (12.2) and 57.7 (19.5) mg/d assessed by one or three 24-h recalls, respectively. Two small studies of Canadian children aged 4–8 y in Ontario (*n* = 41) and Alberta (*n* = 91) assessed DHA intakes over 3 d and reported mean (SD) intakes of 54.1 (72.7) and 37 (58) mg/d DHA, respectively (59, 60). A national dietary survey (24-h recall) of children aged 4–8 y in Australia (*n* = 1216) reported a mean (SD) and median (IQR) of 35.9 (92.7) and 5.1 (0.9–26.5) mg/d DHA, respectively, indicating that at least 75% of the children had DHA intakes below the mean (61). Clearly, the usefulness of dietary assessment of 1 or 3 d may be limited for nutrients that may not be consumed daily, such as DHA. Additionally, this study suggests that the mean or median DHA intake assessed using an FFQ may not reflect the intakes of the majority of individuals in a group and reporting DHA intakes beyond mean and SD or SE data to include distributions or ranges will enable better comparison of DHA intakes within and across populations. An important limitation of this study is that social desirability bias may have influenced the parent's report of their child's dietary intake, which may not accurately represent their actual daily intake.

Associations between DHA intake and RBC fatty acids among the children in the present report were variable, with some children with low intakes having RBC DHA concentratons equivalent to some children with high intakes (Figure 1). Thus, high DHA intakes may protect children from having a low RBC DHA, but an apparent low dietary DHA intake does not necessarily imply low RBC DHA. Other studies have reported moderate relations between dietary DHA and RBC DHA (rho = 0.3–0.6) (58, 62–64). Notably, we found stronger associations between dietary DHA and a potential marker of DHA sufficiency, DHA/22:4ω-6 + 22:5ω-6, than RBC DHA. However, at low DHA intakes, considerable variability was still apparent in the associations between DHA intake and DHA/22:4ω-6 + 22:5ω-6 among the children. This suggests that the majority of variability in RBC DHA status may not be due to dietary DHA alone, highlighting implications for studies using DHA intake as a marker of DHA status. Notably, ethnicity appeared to influence the association between DHA intake and RBC fatty acids (Table 3). In most cases for the ω-3 fatty acids, there was a very small increase in the strength of the association when adjusted for ethnicity, but in the case of 22:4ω-6, the association with DHA intake was substantially reduced. Whether or not these differences may be explained by other dietary factors is unknown, but ethnicity-specific differences in FADS activity have previously been shown (65). Thus, further work is required to determine whether genetic variability may place some individuals at risk for low circulating concentrations of DHA, specifically in individuals who do not consume DHA, and also if variations in fatty acid metabolism genes are related to ethnicity.

For White children, there were significant, but weak, associations between RBC fatty acids and neurodevelopmental tests. In a US study, a positive association between child RBC DHA and language (PPVT) scores at 4 y (*r*^2 ^= 0.14, *P *= 0.018) was reported, with a stronger association when Hispanic/Latino children (major minority group) were removed from the analyses (*r*^2 ^= 0.21, *P *= 0.018). In another study, cheek cell DHA was positively associated with nonverbal IQ (*r* = 0.10, *P *< 0.05) in children aged 8–10 y (34). The latter study also reported positive associations between cheek cell 22:4ω-6, 22:5ω-6, and ω-6 metabolites/ω-3 metabolites with measures of child behavior, including inattention and impulsivity (34).

In the present study, dietary DHA was not associated with neurodevelopment test scores, with the exception of short-term memory (KABC Sequential), which was positively associated with dietary EPA and DHA. Similarly, a US study of children aged 7–9 y reported that total ω-3 fatty acid intake was positively associated with accuracy on hippocampus-dependent relational memory (*r* = 0.28, *P *= 0.04) but not hippocampus-independent memory (*r* = 0.21, *P *= 0.14) (35). Notably, the hippocampus has been suggested to be involved in both short- and long-term memory as well as learning (66), and experimental studies have shown that the structure and function of the hippocampus is altered by an ω-3 fatty acid-deficient diet in development (11, 67–69). In the present study, the KABC Learning scale (long-term information storage and retrieval) was not related to dietary DHA or RBC fatty acids. In contrast, experimental studies have shown inferior learning ability in ω-3 fatty acid-deficient rats (8, 70–73). In animal studies assessing learning ability and ω-3 fatty acids, an ω-3 fatty acid-deficient diet is often given for 2 or more generations; thus, it may be that DHA was not low enough in our population to have an effect on learning. One study in healthy humans reported improved verbal learning ability in children aged 7–9 y after supplementation with DHA (30). However, it is possible that the verbal learning assessment was more related to language development, which has previously been associated with the DHA supply during pregnancy (20, 23, 74, 75), infancy (76), and childhood (31).

To summarize, our results suggest that child RBC DHA is associated with scores on some neurodevelopment tests assessed at 5.75 y, while dietary DHA was only associated with a test of short-term memory, and is consistent with continued brain growth, including synthesis of DHA-rich synapses, throughout the first 6–7 y of life (40). However, the lack of association of dietary DHA with RBC DHA suggests that the associations between RBC fatty acids and neurodevelopmental test scores may not be fully explained by DHA intake, at least not at low DHA intakes. In addition, this study emphasized the importance of dietary methodology to assess DHA intake; mean and median DHA intakes do not reflect the individual intakes of the majority in the group. Although our data suggest that the FFQ provided a better estimate of DHA intake compared with 24-h recalls, the RBC DHA may not be explained by DHA intake alone, with potential ethnicity-specific interactions also noted. This raises the possibility that potential interactions with ethnicity may be overlooked if sample sizes are inadequate to detect differences between ethnic groups. Stronger associations of both dietary DHA and scores on neurodevelopment tests with RBC DHA/22:4ω-6 + 22:5ω-6 than RBC DHA were found, but variability at low DHA intakes was high. Thus, whether RBC DHA/22:4ω-6 + 22:5ω-6 is a more sensitive indicator of DHA sufficiency in the CNS requires further investigation. Finally, although the critical time period(s) when the developing brain is most vulnerable to inadequate DHA is not yet known, this study supports evidence that the child's DHA status may have a role in neural function.

## Supplementary Material

nzac099_Supplemental_File

## Data Availability

Data described in the manuscript, code book, and analytic code will not be made available because we did not obtain informed consent to release participants’ data as the study was conducted prior to 2019.

## Notes

Supported by the Canadian Institutes for Health Research (CIHR) grant number MOP 84248.

Author disclosures: The authors report no conflicts of interest. The funders had no role in study design, data collection and analysis, decision to publish, or preparation of the manuscript.

Supplemental Tables 1–9 are available from the “Supplementary data” link in the online posting of the article and from the same link in the online table of contents at https://academic.oup.com/cdn/.

SMI is deceased.

Abbreviations used. ALA, α-linolenic acid; ARA, arachidonic acid; Beery, Beery–Buktenica Developmental Test of Visual-Motor Integration; CNS, central nervous system; ELOVL, fatty acid elongase; FADS, fatty acid desaturase; FFQ, food-frequency questionnaire; KABC, Kauffman Assessment Battery for Children; LA, linoleic acid; MPI, Mental Performance Index; PPVT, Peabody Picture Vocabulary Test; TOVA, Test of Variables of Attention.
