# Supplementary material for: Complexity of understanding the role of dietary and erythrocyte docosahexaenoic acid (DHA) on the cognitive performance of school-age children
Source: Curr Dev Nutr. 2022 Jun 16;6(7):nzac099. doi: 10.1093/cdn/nzac099 (PMC9283108; doi:10.1093/cdn/nzac099)
Supplement: nzac099_Supplemental_File [file nzac099_supplemental_file.docx]

**Supplemental Table 1.** Dietary intake of ω-3 and ω-6 fatty acids for children in the follow-up or cross-sectional group, assessed using FFQ and one and three 24 h recalls

|  |  | Follow-Up | Cross-Sectional | *P* |
| --- | --- | --- | --- | --- |
| DHA, mg/d | FFQ | 43.2 (1.73 – 300) | 55.6 (4.86 – 336) | 0.075 |
|  | 1 x 24 h recall | 11.0 (0.00– 215) | 14.5 (0.00 – 478) | 0.132 |
|  | 3 x 24 h recalls | 18.1 (0.85 – 274) | 23.0 (1.15 – 381) | 0.358 |
| EPA, mg/d | FFQ | 18.4 (0.58 – 180) | 29.1 (0.82 – 262) | 0.179 |
|  | 1 x 24 h recall | 2.20 (0.00 – 199) | 3.00 (0.00 – 347) | 0.265 |
|  | 3 x 24 h recalls | 3.85 (0.00 – 185) | 4.30 (0.10 – 262) | 0.343 |
| ALA, g/d | FFQ | 1.17 (0.48 – 2.59) | 1.10 (0.53 – 2.97) | 0.973 |
|  | 1 x 24 h recall | 0.85 (0.24 – 2.92) | 0.90 (0.24 – 4.08) | 0.218 |
|  | 3 x 24 h recalls | 0.87 (0.38 – 1.85) | 0.98 (0.39 – 3.36) | 0.180 |
| ARA, mg/d | FFQ | 63.0 (14.4 – 218) | 77.8 (12.4 – 236) | 0.033 |
|  | 1 x 24 h recall | 46.0 (2.22 – 229) | 54.1 (0.60 – 276) | 0.124 |
|  | 3 x 24 h recalls | 51.9 (8.61 – 209) | 67.9 (7.00 – 227) | 0.088 |
| LA, g/d | FFQ | 8.86 (3.64 – 22.3) | 8.71 (3.64 – 19.5) | 0.967 |
|  | 1 x 24 h recall | 7.57 (2.37 – 18.1) | 7.21 (1.94 – 26.0) | 0.865 |
|  | 3 x 24 h recalls | 6.91 (3.22 – 16.8) | 7.34 (2.67 – 18.3) | 0.587 |

ALA, alpha-linolenic acid; ARA, arachidonic acid; DHA, docosahexaenoic acid; EPA, eicosapentaenoic acid; LA, linoleic acid

Data are median (2.5-97.5 percentile) and compared by Mann-Whitney *U* test. For the Follow-up and Cross-Sectional groups, respectively, n = 98, 182 for the FFQ, n = 92, 180 for 1 x 24 h recall, and n = 90, 169 for 3 x 24 h recalls.

**Supplemental Table 2.** RBC fatty acids for all children and for the follow-up and cross-sectional groups.

|  | All |  | Follow-Up |  | Cross-sectional |  |  |
| --- | --- | --- | --- | --- | --- | --- | --- |
|  | n = 245 |  | n = 73 |  | n = 172 |  | *P* |
| **Omega-3 fatty acids** |  |  |  |  |  |  |  |
| DHA | 5.40 ± 1.56 |  | 5.26 ± 1.46 |  | 5.46 ± 1.61 |  | 0.346 |
| 22:5ω-3 | 2.50 ± 0.44 |  | 2.58 ± 0.47 |  | 2.46 ± 0.43 |  | 0.067 |
| 20:5ω-3^1^ | 0.86 ± 0.54 |  | 0.75 ± 0.30 |  | 0.91 ± 0.61 |  | 0.006 |
| 18:3ω-3^1^ | 0.19 ± 0.05 |  | 0.20 ± 0.04 |  | 0.18 ± 0.05 |  | 0.014 |
| **Omega-6 fatty acids** |  |  |  |  |  |  |  |
| 22:5ω-6 | 0.64 ± 0.18 |  | 0.68 ± 0.18 |  | 0.62 ± 0.18 |  | 0.009 |
| 22:4ω-6^1^ | 3.34 ± 0.82 |  | 3.56 ± 0.74 |  | 3.25 ± 0.84 |  | <0.001 |
| 20:4ω-6^1^ | 16.1 ± 1.92 |  | 16.3 ± 1.87 |  | 16.0 ± 1.93 |  | 0.100 |
| 18:2ω-6 | 11.8 ± 1.12 |  | 11.8 ± 0.97 |  | 11.8 ± 1.18 |  | 0.885 |
| DHA/ 22:4ω-6 + 22:5ω-6^1^ | 1.47 ± 0.69 |  | 1.32 ± 0.60 |  | 1.53 ± 0.72 |  | 0.021 |

ALA, alpha-linolenic acid; ARA, arachidonic acid; DHA, docosahexaenoic acid; EPA, eicosapentaenoic acid; LA, linoleic acid

Data are mean ± SD and compared by student t-test

^1^ Data are not normally distributed and compared by Mann-Whitney *U* test. For follow-up and cross-sectional group, respectively, median (2.5-97.5 percentile) RBC 20:5ω-3, 0.68 (0.43-1.55) and 0.73 (0.46-2.70); 18:3ω-3, 0.20 (0.12-0.30) and 0.18 (0.09-0.30); 22:4ω-6, 3.55 (1.90-4.91) and 3.25 (1.70-4.91); 20:4ω-6, 16.6 (10.1-18.7) and 16.3 (11.4-19.1); DHA/ 22:4ω-6 + 22:5ω-6, 1.21 (0.56-3.13) and 1.38 (0.64-3.49).

**Supplemental Table 3.** Development test scores for children in the follow-up or cross-sectional group.

|  | Follow-Up | |  | Cross-sectional | |  |
| --- | --- | --- | --- | --- | --- | --- |
|  | n | Median (2.5-97.5 %) |  | n | Median (2.5-97.5 %) | *P* |
| **PPVT** | 97 | 117 (73.9 – 144) |  | 174 | 115 (73.8 – 146) | 0.185 |
| **Beery** | 98 | 17.0 (12.5 – 21.0) |  | 186 | 17.0 (12.7 – 21.0) | 0.456 |
| **KABC** |  |  |  |  |  |  |
| Sequential | 95 | 22.0 (12.4 – 32.0) |  | 180 | 22.0 (12.5 – 29.0) | 0.082 |
| Learning | 94 | 23.0 (13.4 – 34.0) |  | 176 | 21.0 (12.0 – 31.0) | 0.021 |
| Simultaneous | 98 | 35.0 (18.5 – 44.0) |  | 182 | 35.0 (22.2 – 44.0) | 0.875 |
| MPI | 92 | 81.0 (50.3 – 99.0) |  | 169 | 78.0 (54.2 – 98.8) | 0.026 |
| Delayed Recall | 94 | 22.0 (10.4 – 30.6) |  | 170 | 21.0 (14.0 – 29.7) | 0.047 |

PPVT, Peabody Picture Vocabulary Test; Beery VMI, Beery-Buktenica Developmental Test of Visual-Motor Integration; KABC, Kaufman Assessment Battery for Children, 2^nd^ edition; MPI, Mental Performance Index

Data are compared by Mann-Whitney *U* test.

**Supplemental Table 4.** Macronutrient intake for all children, assessed by FFQ and one and three 24h recalls.

|  | FFQ, n = 280 | |  | 1 x 24 h recall, n = 272 | |  | 3 x 24 h recall, n = 259 | |  |  |
| --- | --- | --- | --- | --- | --- | --- | --- | --- | --- | --- |
|  | Median | 2.5 – 97.5 |  | Median | 2.5 – 97.5 |  | Median | 2.5 – 97.5 |  | *P* |
| Total Energy, kcal/d | 1785 | 1035-3033 |  | 1495 | 848-2563 |  | 1489 | 926-2289 |  | <0.001 |
| Protein, g/d | 70.6 | 41.3-132 |  | 58.9 | 25.8-113 |  | 59.4 | 33.7-103 |  | <0.001 |
| Protein, %energy | 16.0 | 12.0-22.0 |  | 15.6 | 9.27-24.0 |  | 15.9 | 10.9-22.3 |  | 0.444 |
| Carbohydrate, g/d | 244 | 132-417 |  | 195 | 92.7-364 |  | 196 | 113-307 |  | <0.001 |
| Carbohydrate, %energy^1^ | 54.0 | 42.3-65.5 |  | 52.5 | 35.5-70.6 |  | 53.2 | 39.2-66.3 |  | 0.326 |
| Total Fat, g/d | 63.4 | 34.7-119 |  | 55.8 | 18.4-114 |  | 54.0 | 26.6-96.9 |  | <0.001 |
| Total Fat, %energy^1^ | 32.6 | 24.3-42.1 |  | 33.6 | 15.6-50.0 |  | 33.1 | 21.7-44.9 |  | 0.580 |

FFQ, food frequency questionnaire; IQR, interquartile range; 2.5 – 97.5, 2.5 – 97.5 percentile
Data was non-normally distributed and analyzed by Kruskal Wallis test.

^1^Data are normally distributed and analyzed by ANOVA. For FFQ, one and three 24h recalls, mean ± SD for carbohydrate, %energy was 53.9 ± 5.57, 53.2 ± 9.48, and 53.1 ± 6.72, and for total fat, %energy was 32.7 ± 4.47, 33.3 ± 8.52, and 33.2 ± 5.62, respectively.

**Supplemental Table 5.** Associations between child DHA intake and RBC ω-3 and ω-6 fatty acids.

|  | DHA Intake | | | | | | | |
| --- | --- | --- | --- | --- | --- | --- | --- | --- |
|  | FFQ | |  | 1 x 24 h recall | |  | 3 x 24 h recall | |
|  | n = 239 | |  | n = 235 | |  | n = 224 | |
| RBC, % total fatty acids | rho | *P* |  | rho | *P* |  | rho | *P* |
| ω-3 fatty acids |  |  |  |  |  |  |  |  |
| DHA | 0.383 | <0.001 |  | 0.294 | <0.001 |  | 0.357 | <0.001 |
| 22:5ω-3 | -0.190 | 0.003 |  | -0.076 | 0.248 |  | -0.036 | 0.595 |
| 20:5ω-3 | 0.457 | <0.001 |  | 0.325 | <0.001 |  | 0.371 | <0.001 |
| 18:3ω-3 | 0.016 | 0.811 |  | 0.046 | 0.480 |  | 0.067 | 0.316 |
| ω-6 fatty acids |  |  |  |  |  |  |  |  |
| 22:5ω-6 | -0.432 | <0.001 |  | -0.229 | <0.001 |  | -0.286 | <0.001 |
| 22:4ω-6 | -0.444 | <0.001 |  | -0.243 | <0.001 |  | -0.342 | <0.001 |
| 20:4ω-6 | -0.244 | <0.001 |  | -0.088 | 0.177 |  | -0.195 | 0.003 |
| 18:2ω-6 | -0.021 | 0.747 |  | 0.055 | 0.399 |  | 0.072 | 0.286 |
| DHA/ 22:4ω-6 + 22:5ω-6 | 0.517 | <0.001 |  | 0.363 | <0.001 |  | 0.441 | <0.001 |

DHA, docosahexaenoic acid; FFQ, food frequency questionnaire

Data are spearman’s correlation coefficients.

**Supplemental Table 6.** Dietary intake of ω-3 and ω-6 fatty acids for Caucasian and Chinese children.

|  | Caucasian | Chinese |  |
| --- | --- | --- | --- |
|  | n = 186 | n = 44 | *P* |
| mg/d |  |  |  |
| DHA | 48.3 (4.97 – 322) | 87.5 (4.62 – 323) | 0.005 |
| EPA | 24.9 (0.90 – 252) | 46.1 (0.39 – 264) | 0.013 |
| ARA | 63.2 (19.0 – 217) | 97.6 (8.38 – 413) | <0.001 |
| g/d |  |  |  |
| ALA | 1.15 (0.61 – 2.96) | 1.00 (0.33 – 2.01) | 0.091 |
| LA | 8.72 (4.58 – 21.1) | 8.65 (2.78 – 21.1) | 0.105 |

ALA, alpha-linolenic acid; ARA, arachidonic acid; DHA, docosahexaenoic acid; EPA, eicosapentaenoic acid; FFQ, food frequency questionnaire; LA, linoleic acid

Data are median (2.5-97.5 percentile) and compared by Mann-Whitney *U* test.

**Supplemental Table 7.** The RBC ω-3 and ω-6 fatty acids in Caucasian and Chinese children.

|  | Caucasian | Chinese | *P* |
| --- | --- | --- | --- |
|  | n = 166 | n = 39 |  |
| Omega-3 fatty acids |  |  |  |
| DHA | 5.38 ± 1.52 | 6.06 ± 1.42 | 0.013 |
| 22:5ω-3 | 2.55 ± 0.44 | 2.40 ± 0.42 | 0.060 |
| 20:5ω-3^1^ | 0.82 ± 0.41 | 1.02 ± 0.80 | 0.041 |
| 18:3ω-3^1^ | 0.19 ± 0.05 | 0.19 ± 0.06 | 0.720 |
| Omega-6 fatty acids |  |  |  |
| 22:5ω-6 | 0.64 ± 0.18 | 0.61 ± 0.16 | 0.378 |
| 22:4ω-6^1^ | 3.42 ± 0.78 | 3.07 ± 1.07 | 0.001 |
| 20:4ω-6^1^ | 16.2 ± 1.95 | 15.7 ± 1.48 | 0.014 |
| 18:2ω-6 | 11.7 ± 1.16 | 12.3 ± 1.06 | 0.004 |
| DHA/ 22:4ω-6 + 22:5ω-6^1^ | 1.43 ± 0.68 | 1.81 ± 0.74 | <0.001 |

DHA, docosahexaenoic acid

Data are mean ± SD, compared by student t-test.

^1^ Data are not normally distributed, compared by Mann-Whitney *U* test. For Caucasian and Chinese children, respectively, median (IQR) RBC 20:5ω-3, 0.72 (0.60-0.94) and 0.76 (0.67-1.00); 18:3ω-3, 0.19 (0.16-0.22) and 0.18 (0.15-0.22); 22:4ω-6, 3.42 (2.96-3.88) and 3.08 (2.47-3.32); 20:4ω-6, 16.6 (15.5-17.4) and 16.0 (15.1-16.7); DHA/ 22:4ω-6 + 22:5ω-6, 1.25 (0.97-1.64) and 1.73 (1.31-2.13).

**Supplemental Table 8.** Developmental test scores for Caucasian and Chinese children.

|  | Caucasian | Chinese | n | *P* |
| --- | --- | --- | --- | --- |
| PPVT | 121 (112-132) | 108 (90.5-121) | 178, 44 | <0.001 |
| Beery | 16.0 (15.0-18.0) | 17.0 (16.0-19.0) | 190, 44 | 0.004 |
| KABC |  |  |  |  |
| Sequential | 22.0 (18.0-24.0) | 22.0 (19.0-24.0) | 182, 43 | 0.439 |
| Learning | 21.0 (18.0-25.0) | 23.0 (20.2-27.8) | 179, 44 | 0.022 |
| Simultaneous | 35.0 (32.0-38.0) | 37.5 (34.0-39.8) | 188, 44 | 0.009 |
| MPI | 80.0 (71.0-86.0) | 82.5 (75.0-88.0) | 174, 42 | 0.050 |
| Delayed Recall | 21.0 (18.2-24.0) | 22.0 (19.0-24.0) | 176, 43 | 0.859 |

Beery VMI, Beery-Buktenica Developmental Test of Visual-Motor Integration; IQR, inter-quartile range; KABC, Kaufman Assessment Battery for Children, 2^nd^ edition; MPI, Mental Performance Index; PPVT, Peabody Picture Vocabulary Test

Data are median (IQR), analyzed by Mann-Whitney *U* Test.

**Supplemental Table 9.** Dietary ω-3 and ω-6 fatty acids by quintiles of KABC-II Sequential Scale scores for Caucasian children.

|  | Quintiles of KABC-II Sequential Scale Score | | | | |  |
| --- | --- | --- | --- | --- | --- | --- |
| min-max | 9-17 | 18-20 | 21-22 | 23-24 | 25-34 |  |
| Dietary Fatty acids | n = 38 | n = 26 | n = 36 | n = 38 | n = 38 | *P* |
| DHA, mg/d | 23.8 (13.3-51.0) | 60.2 (27.8-88.3) | 62.3 (24.4-106) | 41.8 (21.4-86.9) | 69.8 (37.9-134) | 0.001 |
| EPA, mg/d | 6.70 (2.60-30.7) | 31.7 (10.2-54.3) | 32.1 (9.90-68.4) | 14.3 (4.58-63.9) | 40.6 (18.2-82.1) | <0.001 |
| ARA, mg/d | 59.3 (43.4-93.6) | 66.6 (51.1-84.6) | 70.8 (49.7-104) | 55.3 (41.2-88.4) | 65.4 (52.6-105) | 0.196 |

ARA, arachidonic acid; DHA, docosahexaenoic acid; EPA, eicosapentaenoic acid; KABC-II, Kaufman Assessment Battery for Children 2^nd^ edition

Data are median (IQR) and quintile 1 and 5 were compared by Mann-Whitney *U* test
